# ASO Author Reflections: Multi-Modal Prehabilitation and Outcomes After Gastroesophageal Cancer Surgery: A Cohort Study of Pulmonary Complications and Resilience to Major Postoperative Events

**DOI:** 10.1245/s10434-026-20480-z

**Published:** 2026-09-09

**Authors:** David S. Liu, Jesse Turner, Ashley Bigaran

**Affiliations:** 1 Department of Surgery, University of Melbourne, Austin Precinct, Heidelberg, VIC Australia; 2 Upper Gastrointestinal Surgery Unit, Division of Surgery, Anaesthesia and Procedural Medicine, Austin Health, Heidelberg, VIC Australia; 3 Victorian Interventional Research and Trials Unit, Department of Surgery, University of Melbourne, Austin Precinct, Heidelberg, VIC Australia; 4 Division of Cancer Surgery, Peter MacCallum Cancer Centre, Parkville, VIC Australia; 5 Wellness and Supportive Care, Olivia Newton-John Cancer & Wellness Centre, Austin Health, Heidelberg, VIC Australia; 6 School of Exercise and Nutrition Sciences, Deakin University, Burwood, VIC Australia; 7 Clinical Innovations Laboratory, Olivia Newton-John Cancer Research Institute, Heidelberg, VIC Australia

## Past

Esophago-gastric cancer surgery and perioperative treatments incur substantial morbidity.^1^ Despite advances in surgical techniques and enhanced recovery after surgery pathways, postoperative complications remain prevalent.^1^ Multimodal prehabilitation including physical, nutritional, psychological, and medical interventions have emerged as bundled mechanisms to improve bio-psychological resilience in those preparing for high-risk esophago-gastric surgery.^2,3^ Its impact on perioperative outcomes was evaluated in this study.

## Present

This single center registry-based trial of 164 participants (control: n = 121, prehabilitation: n = 43) demonstrated that multimodal prehabilitation improved physical fitness and decreased malnutrition rates. This was associated with reduced postoperative respiratory, cardiac, hepatic, and renal complications compared with controls. Additionally, we observed reduced intensive care unit and hospital length of stay and higher rates of adjuvant chemotherapy uptake in those who received prehabilitation. Importantly, these effects were independent of surgical techniques.

## Consumer Reflection

‘Following an oesophageal cancer diagnosis, it is easy to feel defined by illness. This program helped me focus instead on strength, preparation and recovery. It equipped me physically and mentally for surgery, fostered confidence and resilience, and gave me a sense of control during an uncertain time. Most importantly, it reinforced the value of patient-centred, co-designed care in improving both patient outcomes and experience’.

## Future

Given these favorable findings, ensuring equitable access to multimodal prehabilitation should be a priority.^4^ The ongoing deficits in healthcare access for patients in regional and rural areas may be addressed through hybrid models of prehabilitation, including digital delivery.^5^ These models should be evaluated for their feasibility, safety, and efficacy to determine their suitability for reducing this disparity.^6^ Collecting qualitative insights will also be essential for identifying barriers, enablers, and unmet needs, thereby informing the development of new delivery models. Future trials should also aim to determine optimal prescription variables for prehabilitation and explore the implementation of stepped-care models to optimize the use of finite hospital resources.
